# The Limits of Language-Thought Influences Can Be Set by the Constraints of Embodiment

**DOI:** 10.3389/fpsyg.2021.593137

**Published:** 2021-05-06

**Authors:** Prakash Mondal

**Affiliations:** Department of Liberal Arts, Indian Institute of Technology Hyderabad, Sangareddy, India

**Keywords:** language, thought, cogniton, constraints, embodiment, linguistic relativity hypothesis

## Introduction

Language and thought are intimately related to one another, but the level or degree of connectedness between language and thought is not clear due to the fact that the influence of language over thought can be more context-specific or general (see Zlatev and Blomberg, 2015). This reflects general assumptions from the Sapir–Whorf Hypothesis (Whorf, 1956). If the influence of language over thought, thinking, and reasoning is very context-specific in being applicable to specific modes/modalities of cognition, such as color, space, visual motion, etc., this may suggest that the constraints of embodiment determine how modal linguistic symbols come to be grounded in neurally instantiated modality-specific systems (Barsalou, 2008).

## Language Altering Brain Wiring and Language Altering Profiles of Reality

Many cognitive consequences are said to ensue from the language-specific conceptualizations of number, color categories, motion, space, and other categories (see Gentner and Goldin-Meadow, 2003; Levinson, 2003; Casasanto, 2004; Majid et al., 2004; Casasanto and Boroditsky, 2008; Wolff and Holmes, 2011; Lupyan, 2012) and can be traced to the properties of our cognitive organization. For instance, the important notion of knowing (or learning) a language different from the first language we acquire in childhood is linked to the rewiring of the brain (see Bylund and Athanasopoulos, 2017). This particular view emphasizes that learning a new way of talking about time encoded in a language makes the user of the language adopt a new way of thinking that was not available to that language user who was ingrained in a distinct way of thinking encoded in his/her first language. This leads to the conclusion that bilingual people display a kind of cognitive flexibility in being able to juggle multiple ways of thinking modulated by the structures of the particular languages used. What is striking here is the premise upon which this study is based: different languages employ different versions of reality that are, in fact, different ways of organizing the same (or even similar) chunk of experiences. This is the crux of the *linguistic relativity hypothesis* or the *Sapir–Whorf Hypothesis* (Whorf, 1956). The *Sapir–Whorf Hypothesis* has met with criticism (Pinker, 2007; McWhorter, 2014), but Chafe (2018) has defended the hypothesis by showing that language influences thoughts *via* the creation of semantic structures distilled from the real-world experiences. The goal here is not to capture this debate. Rather, this paper will point out that the special role of natural language in charting out the territory of cognition must be explored with caution and, if needed, suspicion, especially when the entry is through particular languages.

The problem can be illustrated with some simple cases classified as “context-specific” influences of language over thoughts by Zlatev and Blomberg (2015). For instance, Slobin (2003) in his study of motion verbs in languages including Spanish and English concludes that thoughts about motion are determined by the way languages encode conceptualizations of motion. Thus, languages like Spanish incorporate the conceptualization of path in motion verbs, whereas languages like English incorporate manner in verbs of motion (such as “slide” or “roll”), and this is assumed to induce Spanish speakers to visually interpret path more easily, or conversely, to induce English speakers to tend to fall into a salient visual interpretation of manner. This seems to be a kind of motion warp in the mind, much like the time warp discussed by Bylund and Athanasopoulos (2017). The view that the specific languages we speak influence *and* determine the thoughts we have and entertain appears to fix the point for entry into the domain of humanly realized thoughts and reasoning. However, this is misleading on several grounds. First, the lens-like nature of specific languages permitting differences in thoughts and reasoning that take the form of differentially perceived realities is itself a thought. Furthermore, it is not clear why we should be disposed to think it is language rather than, say, the human memory or even the human competence for social cognition that can influence and determine thoughts and reasoning. After all, it is important to understand that the human memory or the human competence for social cognition is also unique in humans, and also that language itself is an aspect of cognition and cannot be divorced from it (Chomsky, 1993).

Second, the central motivation is that we can gain entry into the territory of human thoughts and reasoning by examining the structures of specific languages. This supposition risks taking language to be *the* entry point rather than *an* entry point for the exploration of human thoughts and reasoning. Language-specific conceptualizations may also be at loggerheads with the dictates of our cognitive organization. It may be observed that the manner of motion and the path of motion may have contextually grounded salience effects in our visual encounters in day-to-day life. These effects are in part due to the nature of our conceptualizations of the manner of motion and the path of motion, and in part due to the properties of body–world interactions engaging with physical events and motions out there in the world (Northoff, 2018). Thus, for example, if a baby is found by her parents to be crawling under a table, it is the path of the motion that may be more perceptually salient than the manner of motion. That is because crawling is what babies usually do (unless an aberrant behavior in crawling is discovered in babies). However, if a car comes hurtling round the corner, the manner of motion of the car rather than the exact path of motion of the car may be more perceptually salient for someone standing nearby. Significantly, it is noteworthy that the manner of motion may be, at least in most pictures, paintings, or images, as perceptually salient as the path of motion since both the manner of motion and the path of motion become abstractions that have to be inferred from the static representations of dynamic events anyway. However, the manner of motion is by its very nature more dynamic than the path of motion unless, of course, paintings or pictures are created to generate a perceptual bias in favor of either the manner of motion or the path of motion. Hence, the interactions with the outer world can help determine the effects of perceptual salience in many cases. Even if language users may be induced to use a particular type of linguistic salience effect, it does not follow that the language-based conceptualizations *cause* language users to saliently use one or the other sort of conceptualization when they use specific languages in a task, say, the reporting of mental imagery (Mondal, 2017).

Learning a new way of talking about time encoded in a language may not make the user of the language adopt a new way of thinking. Rather, new ways of thinking about time (such as a vertical strategy of thinking about time in Chinese along with the horizontal way) may already be as cognitively salient as the old ones because they are abstractions from lived experiences with the ongoing events and actions. They have to be inferred too. Moreover, it is quite plausible that the *actual* conceptualizations of ways of thinking about time constructed during the language users' engagement in linguistic tasks are equally salient in their minds, and it is the linguistic expressions produced that appear to be rough markers or paraphrases of the actual conceptualizations. This winds up conveying the impression that the underlying cognitive representations are determined by the relevant properties of particular languages. That is because language users have no way other than that of producing the specific linguistic expressions their languages admit of. This may have nothing whatever to do with the actual and exact forms of thinking strategies for time. The “calibration problem” between categories of language and categories of thought remains, because categories of thought can have an independent realm (Lucy, 1992). This is certainly not to deny that language-based conceptualizations of a particular strategy for time exist in language speakers' mental repertories, for the influence of language over thoughts cannot be outright ignored (Zlatev and Blomberg, 2015). After all, certain thoughts may be easily accessible and expressible in a language (especially in vocabulary) *via* the interface between syntax/phonology and meaning (Jackendoff, 2002). Rather, this is to reject the idea that language-based conceptualizations of thinking strategies for time do the whole job when language users engage in the diverse tasks.

## Language and Cognitive Reality

The case for cognitive flexibility in bilinguals can be accounted for in a way that reflects the cognitive reality rather than any linguistic version of reality. Thus, for instance, when bilinguals switch from one way of thinking about time to another while shifting from the context of one language to another, it is not the language that induces the bilinguals to do so. Rather, it is the raw cognitive imprint or the mental signature the word evokes/triggers that induces bilinguals to switch ways of thinking. The observed linguistic effects on cognitive strategies in thinking when using language are stabilized regularities of a fluctuating cognitive system. Evidence for such a stance comes from the fact that the activation of modal semantic features in both brain-damaged patients and normal people is not deterministic but rather dynamically governed by many factors some of which are contextual and some of which are purely cognitive in themselves (Kemmerer, 2019). This is also because the constraints of embodiment determine how modal linguistic symbols come to be grounded in neurally instantiated modality-specific systems (Barsalou, 2008). Thus, the constraints of embodiment are not selectively and exclusively oriented and restricted to language. Rather, the aspects of the cognitive system minus language can project certain modes of thinking. This does not amount to supporting any kind of invariance thesis for language and thought as defended in Dupre (2020) based on the assumed conformity of thoughts to structures generated by the language faculty. Instead, it is variation in thought that is perhaps more pervasive due to the brain–world interactions in linguistic experiences, but this variation need not be explained by variation in languages.

Any word in any language known by a bilingual speaker that can kindle the same cognitive schema (or mental impression) can do an equal job. For example, there is nothing that would prevent English–Spanish bilinguals from looking at the activity of jogging as a whole conceptual unit rather than as running in a slow and steady manner, primarily because that is how it may be contextualized as jogging. Therefore, the cognitive reality hidden beneath languages may be stronger than the linguistic projection of reality, and this could undermine any (specific) language-to-reality mapping. However, this is not to outright deny that there cannot be any projection of linguistic reality. The conceptualization of the word “jihad” in Arabic is such a case. However, effects of this kind are limited to inter-cognitive (from cognition to behavior and all the way to cultural praxis) culturally shaped cognitive intrusions that need not warrant brain rewiring any more than the concept of “quarantine” requires brain rewiring. As for the observed linguistic effects on cognitive strategies in thinking when using language, these effects are stabilized regularities of a fluctuating cognitive system. Why think that the conceptual space of cognition is a fixed system that can be molded by linguistic influences? The cognitive space can itself be a dynamical system that is attracted to aspects of conceptualization targeted by certain words but not others. Thus, it is not the words or constructions that bend conceptual space; rather, the conceptual system itself elastically bends to accommodate various configurations when subtle shades of myriad aspects of conceptualization are involved. The role languages play here is that of a pointer. But then, anything non-linguistic can also be a pointer in more or less the same way. Hence, there is no wonder that the concept of a beautiful musical instrument that a piano is may come to the mind when one hears the sound of music played on a piano, even though no one utters the word “piano.” There can be many windows for entry into the uncharted grand hall of cognition.

## Author Contributions

The author confirms being the sole contributor of this work and has approved it for publication.

## Conflict of Interest

The author declares that the research was conducted in the absence of any commercial or financial relationships that could be construed as a potential conflict of interest.
